# Mid-arm circumference method is invalid to estimate the body weight of elderly Emergency Department patients in the Netherlands

**DOI:** 10.1097/MD.0000000000016722

**Published:** 2019-08-09

**Authors:** Marieke H. Opdam, Kristine W.A.C. Koekkoek, Tom Boeije, Nieke Mullaart, Arthur R.H. van Zanten

**Affiliations:** a Emergency Department, Amsterdam University Medical Center, Amsterdam; b Department of Intensive Care Medicine, Gelderse Vallei Hospital, Ede; c Emergency Department, Dijklander Hospital, Hoorn, The Netherlands.

**Keywords:** body weight, elderly, emergency medicine, estimation

## Abstract

In the Emergency Department (ED) actual body weight (ABW) is essential for accurate drug dosing. Frequently, the ABW is unknown and direct measurement troublesome. A method using the mid-arm circumference (MAC) to estimate ABW has been developed and validated in the United States of America (USA). This study aimed to validate the MAC-formula for estimating ABW in the Dutch population and compare its performance within the American population.

Data were obtained from the Dutch National Institute for Public Health and the Environment (RIVM) and extracted from the American National Health and Nutrition Examination Survey (NHANES) datasets. We included all subjects’ ≥70 years whose MAC and weight were recorded and obtained additional anthropometric data. We used the equation: kg = 4 × MAC-50 to estimate the ABW of all subjects and compared results.

We retrieved 723 and 972 subjects from the Dutch and American dataset, respectively. The MAC is better correlated with ABW in the American dataset when compared with the Dutch dataset (Pearson *r* = 0.84 and 0.68, respectively). Bland-Altman bias was –7.49 kg (Limits-of-Agreement [LOA] –27.5 to 12.27 kg) and –0.50 kg (LOA –20.99 to 19.99 kg) in the Dutch and American datasets, respectively.

The MAC based formula to estimate ABW is a promising tool for the elderly American population. However it is not accurate within the Dutch elderly ED population. Consequently, it is not applicable to Dutch EDs. This study highlights that the results of anthropometric studies performed within the USA are not per se generalizable to the European population.

## 1 Introduction

The dosage of certain drugs in the Emergency Department (ED) is based on the actual body weight (ABW) of the patient. Frequently, the ABW is unknown, and for various reasons, direct measurement is troublesome, for example, in the case of hemiplegia or patients that are unconscious or immobilized.

Hospital beds with built-in scales could tackle this problem, yet currently these are not available in many EDs and, consequently, ED physicians are confronted with this clinical problem on a daily basis. Common acute treatments such as fluid regimens in burn patients and procedural sedation and analgesia (PSA) require dosing based on ABW. Inaccurate drug dosing may be hazardous, for example, administering thrombolysis medication for acute ischaemic stroke in dosages too high, increases the risk of fatal intracerebral hemorrhage.^[1,2]^ Therefore, timely and accurate estimation of the ABW is imperative.

Several methods have been developed to estimate ABW in adults, yet, in contrast to acute pediatric care, no universally accepted and validated tool is available for clinicians. In some hospitals, the weight is visually estimated by doctors and paramedics. However, it has been demonstrated that these global estimations are inaccurate.^[3–7]^ In 2007, an anthropometric method was developed to estimate the ABW using a nomogram based on height, waist circumference, and hip circumference.^[8]^ This method appeared to be more accurate than the visual estimation of weight by physicians. However, it was calculated that this method takes 1.5 minutes^[9]^ limiting the feasibility during time-critical situations in the ED. Another study published in 2009 presented a method to estimate ABW using mid-arm circumference (MAC) and height in obese patients.^[10]^ The MAC is measured at the midpoint between the tip of the olecranon and the acromion, with the arm hanging loosely.

Cattermole et al^[11]^ developed a practical method to estimate ABW in adults. The method is based on a simplified formula using the MAC; actual body weight in kg = 4 × MAC (in cm) − 50. This formula is derived from pre-existing datasets from the United States National Health and Nutrition Examination Survey (NHANES) and has been validated in 9022 subjects of another dataset from the same survey. The authors suggested that the simplified MAC formula could be a valuable tool to estimate ABW in adults. Nonetheless, it needs further validation in various clinical settings and among other populations including those in EDs and hospitals in other countries.

This study aimed to validate the MAC-formula for estimating ABW in the elderly Dutch population and comparing its performance within the American population.

## 2 Methods

### 2.1 Data

For this retrospective comparative cohort study we analyzed and compared 2 pre-existing datasets; one from the Netherlands and another from the United States of America (USA). First, we analyzed the food and consumption datasets (FCD) obtained from the Dutch National Institute for Public Health and the Environment (Rijksinstituut voor Volksgezondheid en Milieu-RIVM). The FCD comprises data that has been collected on food consumption and the nutritional status of the Dutch population in general and of specific population subgroups. Within the Dutch FCD database only the elderly cohort (2010–2012) recorded both MAC and ABW and was therefore applicable for further analysis. We included all subjects whose MAC and weight were recorded, and obtained age, sex, height, body mass index (BMI), and abdominal circumference for each subject.

To control for bias we compared our analysis with the publicly available NHANES datasets selecting only subjects of 70 years and older. The datasets were obtained from the Centre for Disease Control and Prevention (CDC) website.^[12]^ We used the NHANES 2009 to 2010 dataset, which was also used by Cattermole et al^[11]^ to validate their formula. We included subjects over the age of 70 years when MAC and weight were recorded. Age, sex, height, BMI, and abdominal circumference were obtained for each subject.

### 2.2 Statistical analysis

Data management and analysis were performed using SPSS V.25 (IBM SPSS statistics for Apple Mac, released 2017, Version 25.0. Armonk, NY). Descriptive data are reported as medians with their interquartile range. Correlation of ABW with various body measurements was determined using Pearson correlation coefficients, *r*, with 95% confidence intervals.

We used the MAC formula; kg = 4 × MAC (in cm) − 50, to estimate the ABW of each subject of the Dutch FCD and American NHANES dataset. To validate this formula, we performed a Bland-Altman analysis^[13]^ and explored accuracy and precision. Accuracy is presented by the Bland-Altman bias, which is the mean difference between estimated and actual weight. Precision is a measure of the spread of estimates around that mean. This is shown by Bland-Altman limits of agreement (LOA), defined as 1.96 × SD, the range within which 95% of the differences between estimated and actual weight will fall.

Also, we determined the level of agreement of the MAC formula with ABW in both datasets using the intraclass correlation coefficient (ICC). An acceptable level of agreement was considered with an ICC >0.7, ICCs of >0.8 and >0.9 were considered good and excellent levels of agreement respectively. Finally, we calculated the proportions of estimates lying within 10, 20, and 30% of the ABW.

### 2.3 Ethical approval

We did not seek ethical approval as the NHANES data was publicly available online and the FCD data was publicly available on request. Furthermore no patient identifiable data was obtained.

## 3 Results

### 3.1 Patient characteristics

A total of 1695 subjects were included. We retrieved 723 subjects (50.6% men) from the Dutch FCD dataset, all aged between 70 and 94 years of age. We extracted 972 subjects (48.5% men) from the NHANES dataset all aged over 70 years. Table 1 depicts the population characteristics of both datasets defined by sex, age, ABW, BMI, MAC, height, and waist circumference.

**Table 1:**
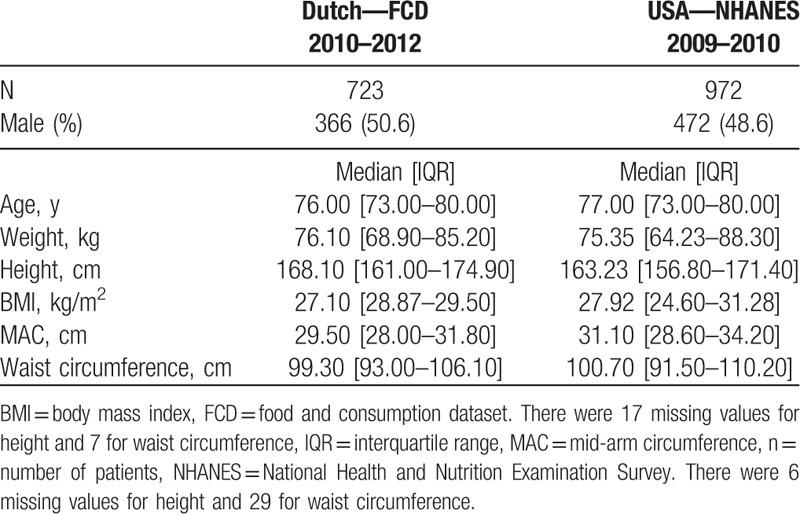
Population characteristics.

### 3.2 Primary outcome

Table 2 shows the correlations of ABW and MAC, height, and waist circumference for both datasets. In the FCD dataset, the waist circumference showed a more substantial positive correlation with ABW (Pearson *r* 0.79, 95% CI 0.76–0.82) than the MAC (Pearson *r* 0.68, 95% CI 0.64–0.71). In the NHANES dataset, both the MAC (Pearson *r* 0.84, 95% CI 0.82–0.86) and waist circumference were strongly correlated with ABW (Pearson *r* 0.91, 95% CI 0.90–0.92).

**Table 2:**
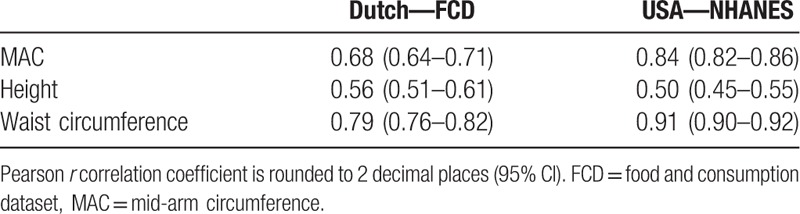
Associations of weight with other body measurements.

**Figure 1:**
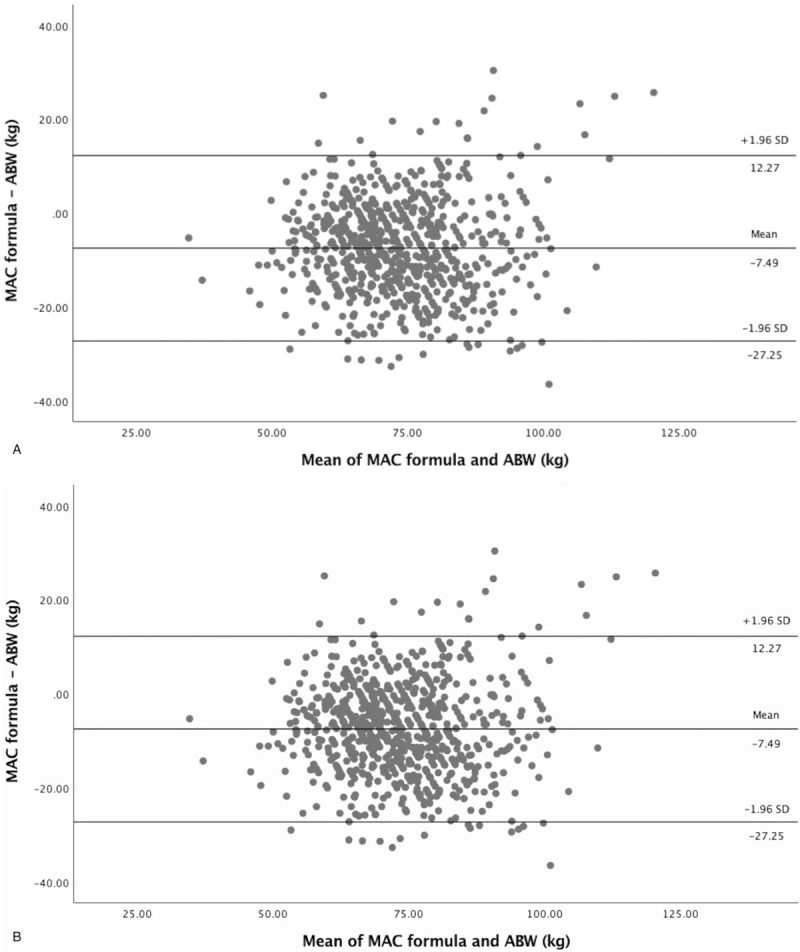
Bland-Altman plots of the mid-arm circumference and actual body weight based on the FCD and NHANES databases, (A) FCD, (B) NHANES, MAC formula = kg = (4 × MAC) − 50. ABW = actual body weight (kg), FCD = food and consumption datasets (Netherlands), MAC = mid-arm circumference, NHANES = National Health and Nutrition Examination Survey (USA).

Bland-Altman results for mean bias and LOA are provided in Table 3, together with the intraclass correlation coefficient and the percentages of estimates ranges within 10, 20, and 30% of the ABW. The Bland-Altman plots are depicted in Fig. 1. In the FCD cohort, the Bland-Altman bias was –7.49 kg and LOA ±9.76 kg. There was an acceptable level of agreement with an ICC of 0.73 (95% CI 0.37–0.86). In the FCD dataset 41.6, 78.0, and 96.3% of the estimates fell, respectively, within 10, 20, and 30% of the ABW.

**Table 3:**
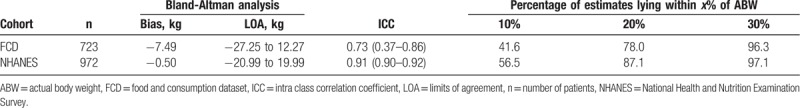
Analysis of weight estimation with the MAC formula.

The Bland-Altman bias of the NHANES cohort was –0.50 and LOA ±0.49 kg. There was an excellent level of agreement with an ICC of 0.91 (0.90–0.92). In the NHANES dataset 56.5, 87.1, and 97.1% of the estimates fell within 10, 20, and 30%, respectively, of the ABW.

## 4 Discussion

Our present comparative cohort study clearly shows that, in a subgroup of elderly patients from the Netherlands and with data derived from the FCD dataset, MAC is poorly associated with ABW. These results are in contrast with the NHANES dataset from the USA. We have demonstrated the accuracy, precision, level of agreement, and goodness of fit of the MAC formula to estimate ABW in elderly cohorts from the Netherlands (FCD) and the USA (NHANES). Our results suggest that this specific formula to estimate ABW based on the MAC is not applicable in the Dutch population of 70 years and older, nevertheless, we were able to show that in the NHANES dataset selecting elderly patients, the performance of the MAC formula was comparable to the data initially published.

We derived the following MAC-based formula for weight estimation from the Dutch dataset; Estimated body weight = 3.33 × MAC – 46.67. To continue with this formula it needs to be validated on a different Dutch dataset. However our results show that in the Dutch population waist circumference has a stronger positive correlation with actual body weight (Pearson *r* 0.79) than MAC (Pearson *r* 0.68), so potentially a waist circumference method might be more suitable for the Dutch population than a MAC method. Nonetheless measuring waist circumference in the ED is impractical and the MAC formula was not explored further.

Our results of the FCD cohort reflect those of Darnis et al who also found that prediction of ABW based on height and MAC was poor.^[14]^ In their study, they addressed the bias and precision of 3 methods that estimate ABW in hospitalized adult patients in Australia. They enrolled 198 patients in 3 hospitals and compared the Lorenz formula (based on height, waist, and hip circumference), the Crandall formula (using height and MAC) and visual estimation of ABW based on the average results obtained by 2 pharmacy interns. Precision was poor in all methods. The Crandall formula estimated ABW within 10% of the ABW in only 67 (34%) patients; error varied between 10 and 20% in 47 (24%) patients and was >20% in 84 (42%) patients. It was concluded that in hospitalized patients, the estimation of ABW by anthropomorphic measures is not accurate.

Moreover, using the Lorenz method within Australian hospitalized patients, only 56% of the patients had ABW estimates within 10% of ABW, which is considerably lower than the results from the Lorenz method validation studies performed in German hospital inpatients admitted with acute stroke, where 94% of the patients had body weight estimates within 10% of ABW.^[8]^ The authors suggested that the Lorenz method might not be generalizable outside of the European stroke patient population where it has been validated.

Cattermole et al^[11]^ found a strong positive correlation between ABW and MAC with a Pearson *r* correlation coefficient of 0.96. Moreover, they presented a Bland-Altman bias of 1.3%, and 56.6 and 84.7% of estimates fell within 10 and 20% of ABW respectively. These results are comparable with the Elderly NHANES cohort. However, it differs from our Dutch FCD cohort. A possible explanation for the conflicting results might be the difference in anthropometry between the elderly American and Dutch populations. The subjects from the Dutch dataset were taller, heavier, and had higher waist circumferences even though the average and median MAC was similar compared with subjects from the USA study population.

Another potential explanation could be differences in body composition and the distribution of fat and muscle mass between the American and Dutch populations. This could be an important issue for further research as this may affect the pharmacokinetics of currently used drugs that are dosed based on ABW and express different volumes of distribution.

A limitation of this study is that it was performed on pre-existing datasets of the general population and performance was not tested in a clinical setting. In the ED, patients presenting with conditions like edema or chronic heart failure would most likely influence the accuracy of the MAC method. However, based on our results we do not recommend validating this formula in daily practice in patients in the ED in the Netherlands. Furthermore, due to the weak correlation between ABW and MAC in the FCD dataset, any formula to estimate ABW using the MAC solely would not be very accurate in the elderly Dutch population. We suggest that before the MAC formula is tested clinically or introduced in other European countries, similar studies to ours could be conducted using available datasets of country-specific populations. It was not possible to assess subjects under the age of 70 in the Dutch population; therefore, it is unknown whether the formula could be used in younger patients in the Netherlands. However, our results have relevance as in the Netherlands 40% of patients presenting to the ED are 70 years or older.^[15]^

In the Dutch dataset 96.3% of estimates using the MAC method were within 30% of the actual body weight, this might indicate that the MAC formula would be suitable to predict the range of body weight of a patient. However, Anglemeyer et al^[7]^ found that the visual estimation of bodyweight of ED patients by attending physicians and residents occurred with an error >20% in 14.7 and 13.4% of the time. The authors believe that physicians working in the ED would be able to visually estimate weights with 30% of the actual body weight and that the use of an additional tool should provide additional accuracy.

This brings us to the most important limitation of this study, which is the absence of evidence or agreement on clinical acceptability of the accuracy that is needed from an estimation tool for ABW. The accuracy that is needed will vary, depending on the type of drug, its therapeutic range and the clinical consequences of under- and overdosing. Proclamations regarding the limits of accuracy for an estimation tool of ABW are outside the scope of this paper, and for comparability, with other studies, we determined proportions of estimates lying within 10, 20, and 30% of the ABW. However to circumvent this critical limitation perhaps future time and effort should focus on the analysis of the cost efficiency of beds with built-in scales and the current managerial challenges that prevent EDs from having them.

This is the first validation of the MAC formula by Cattermole et al^[11]^ in the European and American Elderly population, and the findings of this study suggest that the MAC based formula to estimate ABW is a promising tool for the elderly American population, however it is not accurate within the Dutch population of 70 years and older. Consequently, it is not applicable in the Dutch EDs. This study highlights that the results of anthropometric studies performed on a large group of subjects within the United States are not generalizable to the European population.

## Acknowledgments

The authors are indebted to the Dutch National Institute for Public Health and the Environment (Rijksinstituut voor Volksgezondheid en Milieu-RIVM) for sharing the food and consumption datasets (FCD).

## Author contributions

**Conceptualization:** Marieke Helena Opdam, Tom Boeije, Nieke Mullaart, Arthur R.H. Van Zanten.

**Data curation:** Marieke Helena Opdam.

**Formal analysis:** Marieke Helena Opdam.

**Methodology:** Marieke Helena Opdam, Kristine W.A.C. Koekkoek, Arthur R.H. Van Zanten.

**Project administration:** Marieke Helena Opdam.

**Supervision:** Tom Boeije, Nieke Mullaart, Arthur R.H. Van Zanten.

**Writing – original draft:** Marieke Helena Opdam, Kristine W.A.C. Koekkoek, Arthur R.H. Van Zanten.

**Writing – review & editing:** Marieke Helena Opdam, Kristine W.A.C. Koekkoek, Arthur R.H. Van Zanten.

Arthur RH Van Zanten orcid: 0000-0001-6276-7192.
